# The Ohio Law Regarding the Practice of Medicine

**Published:** 1868-11

**Authors:** 


The Ohio Law Regarding the Practice of Medicine.—We printed the law enacted by our State last winter in the June number of this journal. The law, in most respects, is a good one, and the profession is under obligations to its author, Dr. Kemp. It provides, that to practice medicine in Ohio a man must be a graduate, or must have practiced hitherto for ten years, or produce the certificate of his qualifications to practice, from the State or some County Medical Society. Persons coming into the State, either as permanent or transient practitioners of medicine or surgery, and who do not comply with this law, are liable, on conviction of its violation, to a fine of fifty to one hundred dollars for the first offense, and for the second, a like fine and imprisonment in the county jail for thirty days. This law went into operation on the 1st of October, ult. The only weak place in the law is the permission to Medical Societies to license; and yet, as a beginning, very good progress is made in the right direction; and we scarcely see how this concession could have been avoided to begin with. But it is evident that this permit to license may be so abused, as almost to nullify the utility of the law, and hence it becomes medical societies to seriously consider their duty in the premises.—*Cincinnati Lancet and Observer.*

